# Multi-Source Domain Adaptation Techniques for Mitigating Batch Effects: A Comparative Study

**DOI:** 10.3389/fninf.2022.805117

**Published:** 2022-04-20

**Authors:** Rohan Panda, Sunil Vasu Kalmady, Russell Greiner

**Affiliations:** ^1^Electrical and Computer Engineering, Carnegie Mellon University, Pittsburgh, PA, United States; ^2^Canadian VIGOUR Centre, University of Alberta, Edmonton, AB, Canada; ^3^Department of Computing Science, University of Alberta, Edmonton, AB, Canada; ^4^Alberta Machine Intelligence Institute, Edmonton, AB, Canada; ^5^Department of Psychiatry, University of Alberta, Edmonton, AB, Canada

**Keywords:** resting-state fMRI, multi-source domain adaptation, batch effects, deep learning, ADHD, ASD

## Abstract

The past decade has seen an increasing number of applications of deep learning (DL) techniques to biomedical fields, especially in neuroimaging-based analysis. Such DL-based methods are generally data-intensive and require a large number of training instances, which might be infeasible to acquire from a single acquisition site, especially for data, such as fMRI scans, due to the time and costs that they demand. We can attempt to address this issue by combining fMRI data from various sites, thereby creating a bigger heterogeneous dataset. Unfortunately, the inherent differences in the combined data, known as batch effects, often hamper learning a model. To mitigate this issue, techniques such as multi-source domain adaptation [Multi-source Domain Adversarial Networks (MSDA)] aim at learning an effective classification function that uses (learned) domain-invariant latent features. This article analyzes and compares the performance of various popular MSDA methods [MDAN, Domain AggRegation Networks (DARN), Multi-Domain Matching Networks (MDMN), and Moment Matching for MSDA (M^3^SDA)] at predicting different labels (illness, age, and sex) of images from two public rs-fMRI datasets: ABIDE 1and ADHD-200. It also evaluates the impact of various conditions such as class imbalance, the number of sites along with a comparison of the degree of adaptation of each of the methods, thereby presenting the effectiveness of MSDA models in neuroimaging-based applications.

## 1. Introduction

### 1.1. Motivation and Background

With recent developments in brain imaging technology, data in the form of functional Magnetic Resonance Imaging (fMRI), electroencephalography (EEG), and Magnetoencephalography (MEG) have become widely available, which can be helpful in conducting various diagnostic and predictive analyses. Owing to the spatio-temporal nature of fMRI data, which allows for extensive information extraction, there has been a steady rise in the applications of various deep learning (DL) strategies applied to fMRI data to classify or predict mental illnesses (e.g., Alzheimer's, ADHD, Schizophrenia, etc.), brain states (e.g., sleep stages, task-based activity, etc.), or patient demographics (e.g., age, gender, IQ, etc.).

Deep learning models are data-intensive in nature and tend to work better as we increase the size of the data available for training. However, owing to the difficulties related to the acquisition of fMRI data, building a large dataset is often infeasible, expensive, and time-consuming.

A general workaround involves building a large dataset by combining data from various acquisition sites for a particular research task. This, however, leads to another problem that arises as the data was collected from multiple sites, which means this can involve different acquisition methods, equipment, demographic of patients, methodology, etc. The models can be trained on a dataset that simply contains all of these instances, without any modifications. However, this method ignores these differences that might hamper the model's generalizability (Jiménez-Guarneros and Gómez-Gil, 2020). The basic reason for such variations is the differences in the probability distributions of data and labels across sites, which are generally termed as domain shift, and also, batch effects (Dundar et al., 2007).

Recent studies in various domains have focused on developing methods to mitigate such issues, including domain adaptation (DA) techniques, which aim at building a generalized model that can learn from the multiple given source sites to produce a model that can perform reasonably well on a new, yet related, target site. The existing DA techniques have varied approaches based on factors the number of source sites (single-source DA, multi-source DA), label availability in the target domain (unsupervised, semi-supervised, supervised DA), and method of DA (discrepancy, adversarial, and reconstruction based). Which technique performs best can depend on the objective at hand and the type of datasets that have been used. The purpose of this study is to examine existing DA methods and their performance when dealing with multi-site biomedical (in this case, resting-state fMRI) data.

### 1.2. Related Studies

In the past decade, there have been various new techniques that apply DL tools to fMRI data, to develop predictive models based on numerous objectives.

Many systems view raw fMRI data as a sequence of 3-dimensional data, motivating various techniques which use 3D convolutions to build models such as using 3D-CNN to predict [R3]attention deficit hyperactivity disorder (ADHD) using fMRI and structural MRI (Zou et al., 2017), extracting features using 3D-Convolutional Autoencoders for mild Traumatic Brain Injury recognition (Zhao et al., 2017), predicting Schizophrenia using 3D-CNN, development of 2-channel 3D DNN for autism spectrum disorder (ASD) classification (Li et al., 2018b). Though such methods allow for maximal information extraction, the deep models are computationally very expensive and generally infeasible.

To mitigate this issue, functional connectivity matrices (Lynall et al., 2010) are popularly used and are found to be a good replacement, making the training computationally feasible and also providing a way to interpret the results. Some noteworthy results using FCMs include classification of patients with Schizophrenia using various DL methods (Shen et al., 2010; Arbabshirani et al., 2013; Yan et al., 2017), prediction of other illnesses such as ADHD (Riaz et al., 2020), Alzheimer's (Ju et al., 2017), ASD (Li et al., 2018a; Saeed et al., 2019), and Mild Cognitive Impairment (Chen et al., 2016). There have also been classifications of other brain states, such as suicidal behavior (Gosnell et al., 2019), chronic pain (Santana et al., 2019), migraine (Chong et al., 2017), and demographics such as age (Pruett Jr et al., 2015) or gender (Fan et al., 2020).

While the studies mentioned above have shown impressive results, none addressed the issue of batch effects. However, there have been few recent methods that have tried to deal with batch effects in different ways. Olivetti et al. (2012) were one of the first to investigate batch effects in [R3]resting state-fMRI (rs-fMRI) datasets (ADHD-200) using extremely randomized trees along with dissimilarity representation. Vega and Greiner (2018) studied the impact of classical techniques such as covariate, z-score normalization, and whitening on batch effects. Wang et al. (2019) explored ways to use low-Rank DA to reduce existing biases on multi-site fMRI datasets. Recent approaches include transport-based joint distribution alignment (Zhang et al., 2020), federated learning (Li et al., 2020), and conditional autoencoder (Fader Networks) (Pominova et al., 2020).

It is, therefore, useful to have a comparative survey of the performances of various existing MSDA techniques applied to solve the batch effects in multi-site fMRI datasets, to understand the benefits and limitations of DA approaches.

## 2. DA Methods

We define the common objective of multi-source domain adaptation (MSDA) techniques as follows: Given a collection of labeled source-domain data ${𝔇}_{s}\;=\;{\left\{\left(x_{s}^{i},y_{s}^{i}\right)\right\}}_{i=1}^{N_{s}}$ ∀*s*∈{1, …, *S*}, [R2] where S, *N*_*s*_ denote the total number of sites and number of samples from site “s” and a collection of unlabelled target-domain data ${𝔇}_{t}\;=\;{\left\{x_{t}^{i}\right\}}_{i=1}^{N_{t}}$ (where $x_{s}^{i},\;x_{t}^{i}\;\in \;X$ and $y_{s}^{i}\;\in \;Y$), [R2,R3] where *N*_*t*_, X, and Y denote the number of samples from the target site, input data feature space, and label space, respectively, the goal is to build a classifier that can use information from the source domains to help develop models that can perform accurate classifications in the target domain (Zhao et al., 2020). For our experiments, We take one of the *S* domains as the target domain (by convention, this is the domain indexed by *S*) and use the others as source domains (*s*∈{1, …, *S*−1}). Generally, MSDA techniques employ different strategies of transforming the target domain distribution into the source domain distributions to tackle the issue of batch effects. We allow the marginal probability distributions *P*_*X*_ to be different across domains, but [R1] requireaim for the conditional probability distributions *P*_*Y*|*X*_ [R1] remain the sameto become similar after adaptation. Below is a short introduction to the various methods used in our experiments.

**Figure 1:**
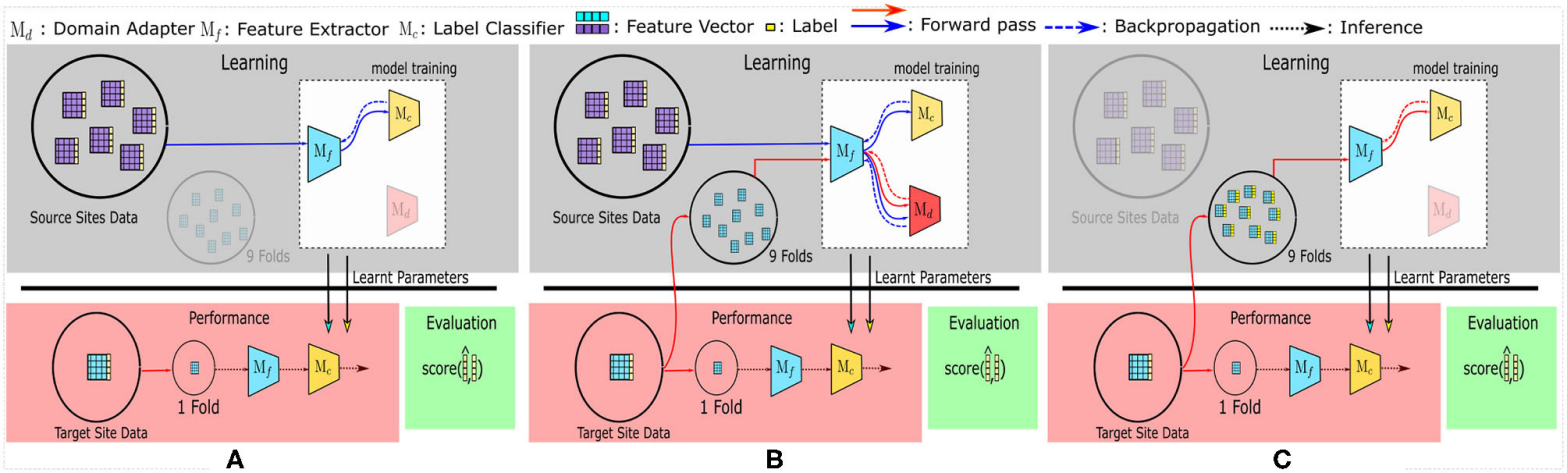
The training and evaluation pipelines used in this study. **(A)** represents the SRC (Source) model, used as the baseline. All other multi-source domain adaptation (MSDA) models can be generalized to be using **(B)**. Finally, to compare the accuracy achieved with target-only data—the TAR model—is shown in **(C)**.

**Domain Adversarial Neural Networks (DANN)**: Considered as one of the fundamental models in DA, DANN (Ajakan et al., 2014) is a single-source DA technique—the only single-source DA method included in the comparison. DANN's architecture is similar to Figure 1B, except that all the sources' data are combined and used as a single big source.

**Multi-source Domain Adversarial Networks (MDAN)**: MDAN can be seen as a natural extension of DANN for MSDA problems. Its feature extractor and label classifier are essentially the same as DANN's, but MDAN uses one domain adapter *M*_*d*_*i*__ for each of the *S*−1 source domains.

Zhao et al. (2018) introduce two versions of MDAN: hard-max and soft-max variants. [R2] The main difference between the two versions is that the hard-max version tries to improve the classification errors which correspond to the worst performing source site while the soft-max version tries to improve errors for all the source sites simultaneously using the log-sum-exp trick (further details in Appendix A). We use the soft-max variant as it is shown to provide better generalization in Zhao et al. (2018).

**Domain AggRegation Networks (DARN)**:

One of MSDA's main challenges is that it needs to include source sites based on the target site, in a way that minimizes the negative transfer while preserving as many training instances as possible. To tackle this issue, DARN (Wen et al., 2020) dynamically selects source sites and gives the sites varied importance based on their label classification losses. This is possible by solving the Lagrangian dual of the objective that needs to be optimized by utilizing binary search strategies.

**Multi-Domain Matching Networks (MDMN)**: MDMN tackles MSDA by first projecting features into a shared feature space. By computing, then using a degree of similarity between the target and source sites, MDMN merges similar sites together to construct the shared feature space, while reducing the negative transfer by keeping dissimilar sites distant. This model tackles this objective by using a loss function based on Wasserstein distance and a special training paradigm as described in Li et al. (2018c).

**Moment Matching for MSDA(M**^**3**^**SDA)**: Unlike the previously discussed models, M^3^SDA aligns target domain with source domains while simultaneously aligning source sites among themselves. Furthermore, it tackles this issue by utilizing the feature distribution moments instead of the raw input features for adaptation, which provides certain robustness and a statistical advantage in MSDA. Peng et al. (2019) introduces an extension of M^3^SDA, called M^3^SDA-β, which they demonstrate performs better against overfitting and provides better generalization. We, therefore, use M3SDA-β to understand the model's performance on neuroimaging data.

Appendix A provides more information about each of these architectures.

## 3. Methodology

### 3.1. Datasets and Tasks

This study uses two different publicly available datasets for training and evaluation, selected on the basis of the number of total scans available, the number of sites of data acquisition, and their frequent usage in the research community.

The first dataset consists of rs-fMRI scans from the ABIDE 1dataset (Craddock et al., 2013a), including 530 control instances (tagged as typical controls, TC) and 505 instances collected from subjects suffering from ASD, which have been acquired from 17 different sites. the phenotypic information and pre-processing steps used in the dataset.

The ADHD-200 dataset is our second multi-site fMRI dataset, which has been compiled from 8 different sites and contains 1,516 rs-fMRI scans in total: 842 scans from control subjects, and 674 from subjects who suffer from ADHD.

[R2]These two datasets have been standard datasets used for the analysis of batch effects in public fMRI databases. For each dataset, we run three different classification tasks, using three different labels: (1) the respective mental illnesses, between illness and control samples; and binary classification of two phenotypic labels, (2) sex, and (3) age (old vs. young, with respect to the global median calculated separately for each of the two datasets).

### 3.2. Functional Connectivity Matrix and Feature Extraction

Functional connectivity is defined as the temporal dependency of spatially-remote neuro-physiological events (Van Den Heuvel and Pol, 2010). It computes the level of co-activation between two spatially separate regions of interest (ROIs) in the brain, based on the mean time-series extracted from these ROIs. Each ROI is pre-defined using some atlas or template. Here, we use the Automatic Anatomical Labeling (AAL) atlas (Tzourio-Mazoyer et al., 2002), which partitions the brain into 116 different non-overlapping ROIs.

We then calculate the functional connectivity matrix (FCM) using Pearson's correlation coefficient between each pair of time-series which results in a 116 × 116 matrix. Since the diagonal of this matrix is redundant and the matrix is symmetric, the diagonal is dropped and the upper triangle of the matrix is flattened to finally produce a vector of size 1162 = 6,670 features for each rs-fMRI scan, which is used as the input data to various models in this study.

### 3.3. Training and Testing Settings

The MSDA models require labeled data flowing in from multiple source domains and a batch of unlabeled data from the target domain. To accommodate this, we first take a single site as the target domain and consider the remaining sites as different source domains. The target domain is then split using a stratified 10-fold CV strategy, wherein a single fold is kept aside for testing while the remaining 9 folds are used (without their labels) to provide the unlabeled target domain data required for the unsupervised-MSDA methods. The folds are kept consistent for the experiments pertaining to each dataset. All data points from the source sites are fed into the model along with their labels during training. We repeat the training and testing for each fold and each site, then report the average accuracies as the results. Figure 1B shows this pipeline.

To set the hyperparameters μ and γ [R1] (refer to Appendix A for their usage), the portion of data used for training (Source domains, along with 9 folds of unlabelled target domain data) is utilized. [R2] The training subset (i.e., data from the 9 folds) of the target domain is divided into 80-20% train-test split, where 80% of the target domain samples along with the source site data is used for searching hyperparameters and 20% of the target site data (with their labels) is used for validation of the selected hyperparameters during its fine-tuning. It is noted that all of the samples used in hyperparameter tuning belong to the training subset of the entire dataset.hyper-param tuning

A total of 30 random samples of hyperparameters are sampled from a wide range of values and used for the tuning process. This tuning occurs once per target domain. The learning rate for the domain adapter and the label classifier is kept constant at 1*10^−4^ when the model is trained on ABIDE 1and at 3*10^−4^ for ADHD-200, respectively. The learning rates are found using a grid search which follows a similar strategy as used for μ and σ.

To compare the performance of MSDA models (Figure 1B), the SRC [R1,R2] (source) model (refer to Figure 1A) is used as the baseline model. [R1]In this setting, data from all the source sites are combined and are treated as one big dataset; i.e., no target site data is used during training.SRC model description Also, the TAR [R1,R2](target) model [R1]uses only the (labeled) target site data (and no source sites)TAR model description, in a stratified [R2]10-fold cross-validation (CV) setting to maintain the class distribution in all the folds. [R1]These models show the baseline performances in two different cases which can be considered as naive approaches to using multi-source datasets. The SRC model tries to use the labeled data from all source sites without considering batch effects or any data from the target site, while the TAR model depicts a model's performance if the training is conducted only on a small labeled subset of the target data, without utilizing any other source site's data. The mean accuracies for SRC and TAR are calculated similarly to the process used for MSDA models, wherein the average of the accuracy scores of the test splits is used. Each of the 10 folds is taken as the test fold iteratively and the final reported accuracy is the average of the 10 accuracy scores. The folds were kept consistent across experiments for each of the datasets respectively.

### 3.4. Model Specifications

The architecture of the various components in each of the pipelines was kept constant, i.e., the feature extractor, label classifier, and domain adapter had the same design across all methods. Since the features were flattened FCM, fully connected layers (FCN) were used along with dropouts and L-2 regularization. The sub-models' designs are described in Table 1. In most cases, the complete model is trained end-to-end using the Adam optimizer (Kingma and Ba, 2014) on the loss functions defined in Appendix A. Few of the models (e.g., MDMN) utilize a training strategy unlike other methods (refer to Appendix A). In such methods, the training process described in the respective original articles is utilized.

**Table 1:** Fully connected layers (FCN) architectures used in each of the sub-models.

**Sub-Model**	**Architecture**	**Output**
Feature Extractor (M_*f*_)	input → 2000 → 1000	latent features
Label Classifier (M_*c*_)	1000 → 100 → 2	label predictions
Domain Adapter (M_*d*_)	1000 → 100 → *n*	site predictions

*Each layer was followed by an Rectified Linear Unit (ReLU) layer (except the last layer where softmax is used) and a dropout layer with p = 0.5. The output of M_d_ has a different number of nodes for different models and datasets and, therefore, is represented by “n”*.

## 4. Data Demographics

The ABIDE 1dataset (Craddock et al., 2013a) is a combination of fMRI scans from 17 different sites. The dataset provides users with rs-fMRI, T1 structural brain images, and phenotypic information for each patient. It consists of 505 ASD scans and 530 controls. As a part of pre-processing the rs-fMRI data, the C-PAC processing pipeline offered by Preprocessed Connectome Project (Craddock et al., 2013b) was used. The pipeline consists of several steps such as slice-time correction, motion correction, intensity normalization, and nuisance signal removal. Furthermore, data from all the sites were spatially registered to the MNI152 template space, along with being passed through a band-pass filter (0.01–0.1 Hz) to remove any high frequency noise in the data The site-wise distribution of age and sex is described in Table 2 Similarly, the ADHD-200dataset (Bellec et al., 2017), which was first introduced during the ADHD-200Competition, contains scans from eight different sites which includes a total of 973 individuals. This dataset also provides one or more rs-fMRI, T1 structural MRI, and the respective phenotype for each individual.The scans undergo similar preprocessing using a pipeline made available by Neuroimaging Analysis Kit (NIAK) which includes steps such as Slice timing correction, motion correction, linear and non-linear spatial normalization, correction of physiological noise, Spatial smoothing, and MNI T1 space registration. The distribution of the data according to the phenotype is provided in Table 3. Since the phenotypic data had inconsistent and missing age information, the particular column has been omitted from the table.

**Table 2:** ABIDE 1demographics.

	**ASD**		**TC**	
**Sites**	**Age**	**Sex**	**Age**	**Sex**
Pitt	19.0 (7.3)	M 25, F 4	18.9 (6.6)	M 23, F 4
Olin	16.5 (3.4)	M 16, F 3	16.7 (3.6)	M 13, F 2
OHSU	11.4 (2.2)	M 12, F 0	10.1 (1.1)	M 14, F 0
SDSU	14.7 (1.8)	M 13, F 1	14.2 (1.9)	M 16, F 6
Trinity	16.8 (3.2)	M 22, F 0	17.1 (3.8)	M 25, F 0
UM	13.2 (2.4)	M 57, F 9	14.8 (3.6)	M 56, F 18
USM	23.5 (8.3)	M 46, F 0	21.3 (8.4)	M 25, F 0
Yale	12.7 (3.0)	M 20, F 8	12.7 (2.8)	M 20, F 8
CMU	26.4 (5.8)	M 11, F 3	26.8 (5.7)	M 10, F 3
Leuven	17.8 (5.0)	M 26, F 3	18.2 (5.1)	M 29, F 5
KKI	10.0 (1.4)	M 16, F 4	10.0 (1.2)	M 20, F 8
NYU	14.7 (7.1)	M 65, F 10	15.7 (6.2)	M 74, F 26
Stanford	10.0 (1.6)	M 15, F 4	10.0 (1.6)	M 16, F 4
UCLA	13.0 (2.5)	M 48, F 6	13.0 (1.9)	M 38, F 6
Maxmun	26.1 (14.9)	M 21, F 3	24.6 (8.8)	M 27, F 1
Caltech	27.4 (10.3)	M 15, F 4	28.0 (10.9)	M 14, F 4
SBL	35.0 (10.4)	M 15, F 0	33.7 (6.6)	M 15, F 0

*The age is represented by the mean (standard deviation) format and the sex distribution is denoted by M: males and F: females*.

**Table 3:** ADHD-200demographics.

	**ADHD**	**TC**
**Site**	**Sex**	**Sex**
KKI	M 15 F 10	M 41 F 28
NINeuroImage	M 31 F 5	M 12 F 25
NYU	M 117 F 34	M 56 F 55
OHSU	M 30 F 13	M 30 F 40
Peking	M 92 F 10	M 84 F 59
Pittsburg	M 3 F 1	M 50 F 44
UWash	M 0 F 0	M 33 F 28
Brown	M 0 F 0	M 9 F 17

## 5. Results

**Figure 2:**
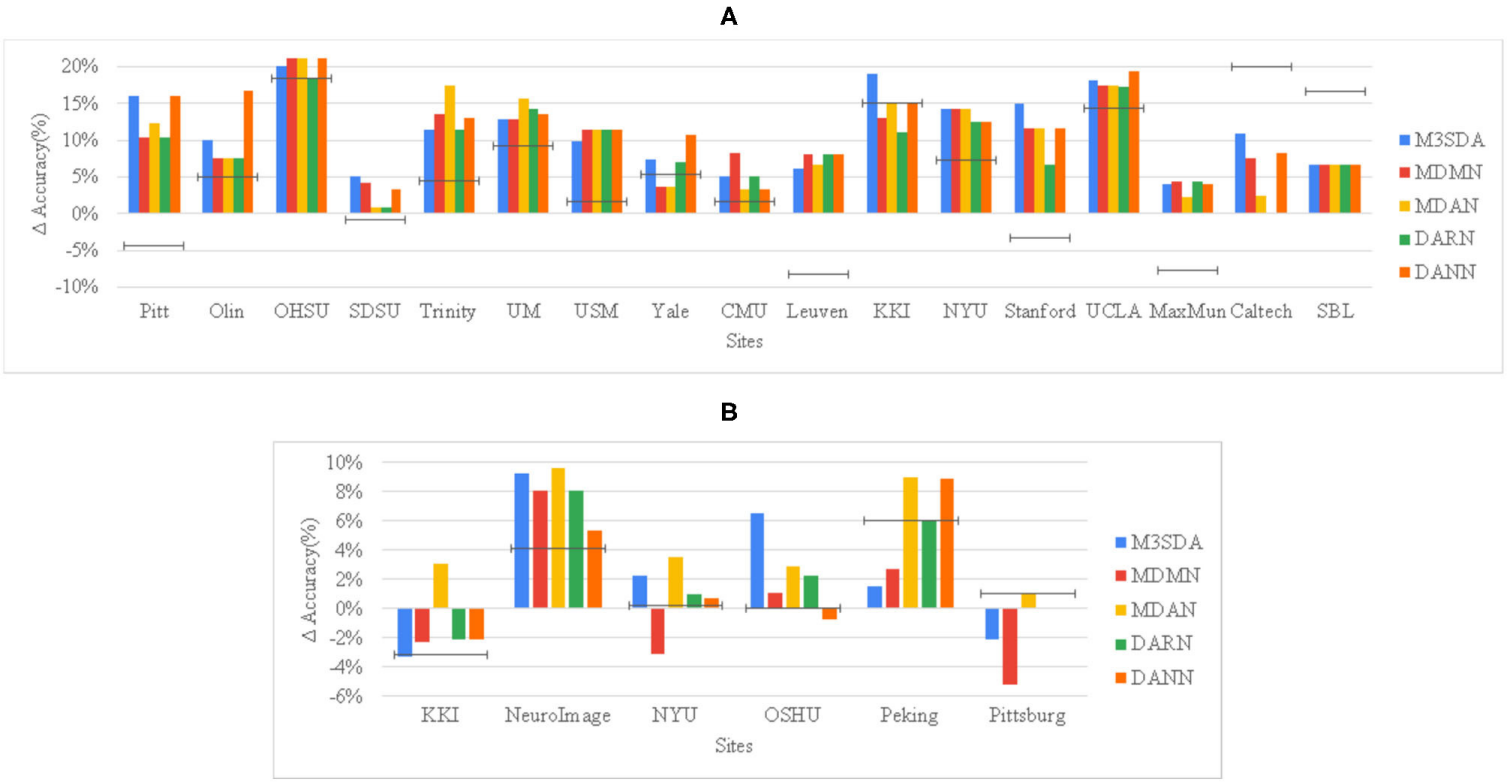
Site-wise performance of MSDA and baseline models in illness classification for ABIDE 1 **(A)** and ADHD-200 **(B)** The height of each bar is the average of 10-fold CV accuracies achieved when the particular site was chosen as the target site. To show how much better an MSDA model performs with respect to using only the target site data, and also as compared to using all source data without any domain adaptation (DA), we then subtract the TAR model accuracy from every model's original accuracy, and show the SRC model's scores as a black target-line.

**Figure 3:**
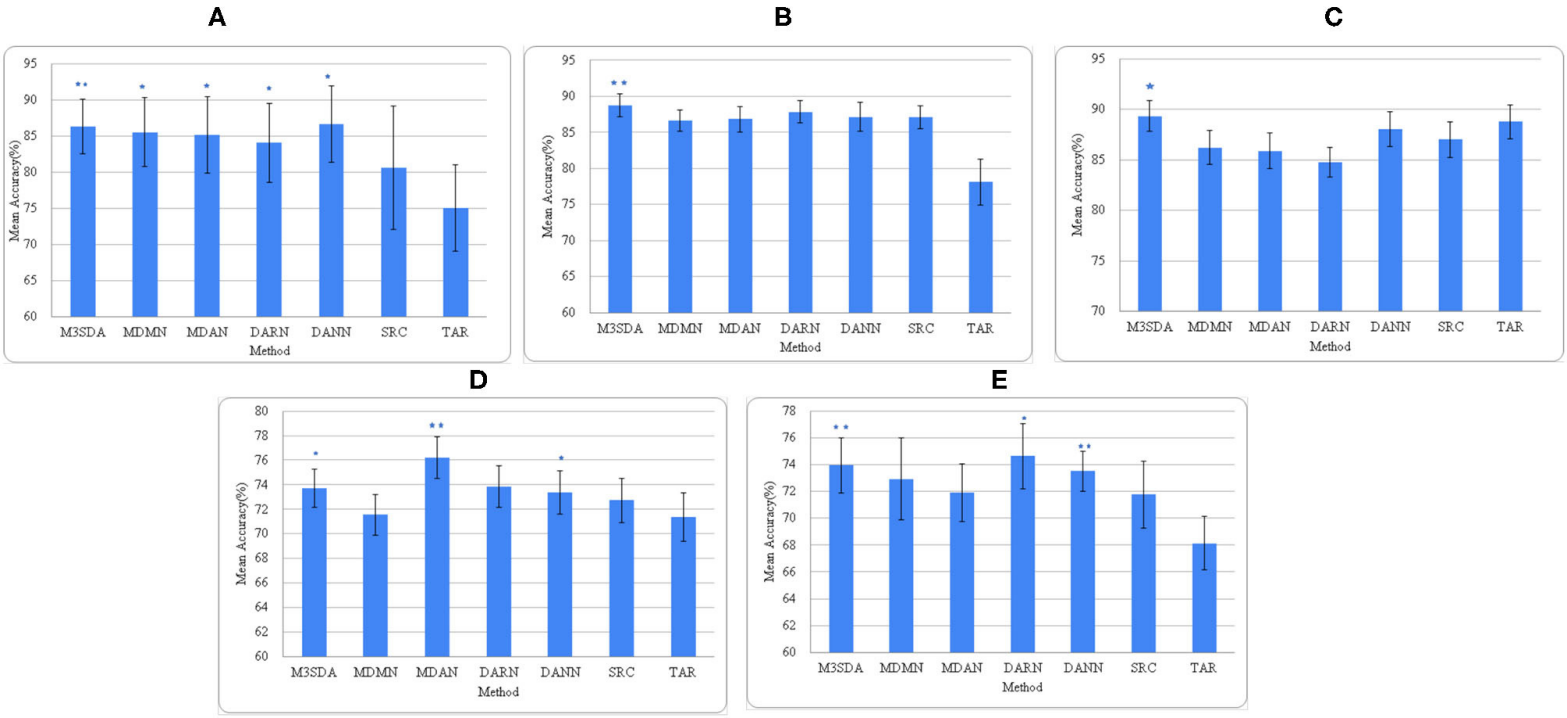
Average accuracies of all models across all sites. **(A–C)** depict the accuracies for illness, age, and sex for ABIDE 1 respectively, while **(D,E)** are for illness and sex for the ADHD-200 dataset. *(p < 0.05) and **(p < 0.01) depict the statistical significance between the MSDA methods and SRC models, each calculated using a paired *t*-test.

**Mental illness**: Figures 2, 3 show that MSDA models produce classifiers that are more accurate than SRC and TAR. In the case of ABIDE 1, we find a statistically significant increase in the accuracy scores in baseline (SRC) as compared to the MSDA models, with the highest increase being in M^3^SDA (5%, p < 0.01). In the case of ADHD-200 data, since most of the sites had imbalanced classes, the data was balanced (refer to Section 5.1) and used for experimentation. In comparison to ABIDE, MSDA is only slightly more accurate than SRC (1–4%) in the ADHD-200dataset. MDAN (76.21%, p < 0.01) has the highest increase in comparison to baseline (72.72%), while MDMN (71.54%) and DARN(75.62%) do not outperform baseline accuracy significantly (Figure 3D).

Figure 2 provides a deeper look at the site-wise performance of the models. For ABIDE 1, Figure 2A shows that all models performed better than TAR, and most of the models performed better than SRC for the various sites, with only a few exceptions. For sites like Caltech and SBL, no model was able to perform better than SRC, while in some sites such as KKI and Yale, a few models performed worse than SRC. For ADHD-200, Figure 2B shows that in the majority of the sites at least one of the models performs worse than TAR. However, in all of these sites, MDAN is able to perform better in comparison to TAR. DARN and DANN scored just as much as TAR when Pittsburgh was used as the target site, which implies that adding or using data from other sites did not improve the model's performance by any margin.

**Age**: Figure 3B shows that, except for M^3^SDA (p < 0.01), applying DA models does not increase the classification performance over the baseline results. The baseline and MSDA models both have an accuracy of around 86–88%. To explore whether this was due to class imbalance, we applied a strategy similar to the one in Section 5.1, however, that did not improve the results.

**Sex**: Figure 3C presents the accuracy scores of the experiments on the ABIDE 1 data. While only M^3^SDA showed a statistically significant increase for ABIDE 1(p < 0.05), in ADHD-200 all the DA models except MDMN and MDAN scored significantly better than baseline scores (Figure 3E). While DARN performs the best and has a higher mean, its significance is weaker in ADHD-200, and it is not so accurate when it comes to ABIDE 1; moreover, we see that M^3^SDA is consistently accurate in both datasets.

### 5.1. Class Balancing

**Figure 4:**
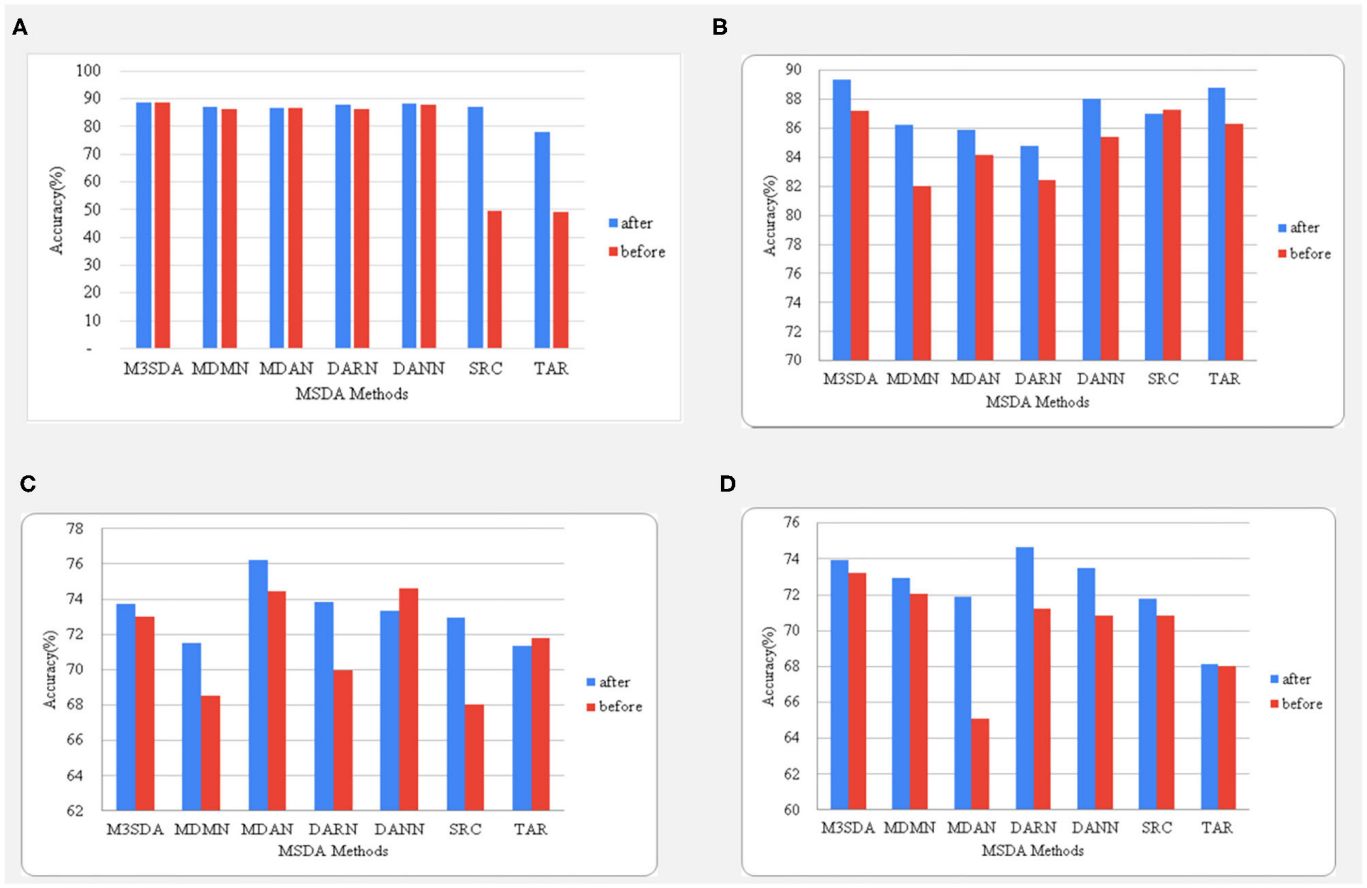
A comparison between the accuracies before and after balancing the classes using oversampling. The age and sex classifications for ABIDE 1 are shown in **(A,B)**, while **(C,D)** represent the illness and sex classifications for the ADHD-200dataset.

As mentioned earlier, to handle the data imbalance the minority class was over-sampled to match in number with the majority class in the training set of the data. [R1] This comprised of randomly sampling data points from the minority class until there is almost an equal amount of samples from both classes. We perform this oversampling on data from all the source sites which are being used for training in the current iteration. In case a particular site consists of data of only one class, the site is dropped in that experiment (e.g., ADHD illness classification displays 6 sites instead of 8). To understand the impact of class balancing on improving the performance of each model, the comparison of the accuracies before and after data balancing is provided in Figure 4. The data in ABIDE 1 for illness already contained balanced classes and, hence, was omitted. [R1] It is noted that under-sampling of the majority class was also experimented with, but since the datasets are already small in size, under-sampling reduces the number of samples the model gets to train on, which deteriorates its performance.

It is seen in Figure 4 that balancing data in the case of age classification for ABIDE 1made no difference when it came to MSDA performances, while we see a big improvement while using this strategy for sex classification with an increase of as high as 8% for the MDMN model. The accuracy changes in ADHD-200are quite different from what is observed for the ABIDE dataset. We see that in the case of illness classification, all models (except DANN) benefited from the balancing. An interesting observation can be made in sex classification for ADHD-200, wherein the accuracy scores of MDAN on the same test folds increased by almost 7%. Hence, in most cases, data balancing had a positive and significant impact on improving model performance.

## 6. Discussion

The aim of this article is to study the performances of various state-of-the-art MSDA models on classifying fMRI scans for different neurological disorders and demographic labels. Apart from the evidence that MSDA models outperform the baseline scores, we also note certain factors which help the MSDA models improve their performances. We noted a positive impact of balancing the data (Section 5.1) using oversampling for the datasets. The increase in accuracies was more in ADHD-200as compared to the increase in ABIDE 1's performance. This observation suggests that current MSDA models might be impacted by the class balance present in the data used, since the ADHD-200dataset is comprised of more imbalanced data than ABIDE 1, we see the aforementioned differences.

Overall, M^3^SDA is the only method among the MSDA methods applied in this study, that showed statistically significant improvement over baseline consistently in all of our prediction tasks. Hence, based on our multiple prediction results in two large multisite datasets, we recommend M^3^SDA as the first choice for MSDA applications in fMRI datasets. M^3^SDA has higher accuracy in most (15 out of 17) of the sites in ABIDE 1, while in ADHD-200, MDAN seems to perform better with some consistency(5 out of 6). Furthermore, the increase in accuracy using MSDA models differs from ABIDE 1 (16–20%) to ADHD-200 (8–10%).

Based on the results for classification of demographic information, we observe that MSDA models do not perform very well when age label is used, furthermore, compared to illness classification, both age and sex classifications have lower differences between MSDA and baseline performances. This can be an indicator that fMRI scans might not be a suitable input feature for demographic classification. Nevertheless, we can note that models like M^3^SDA were able to consistently perform significantly better for both datasets, as seen in Figure 3, which shows its robustness and versatility.

**Figure 5:**
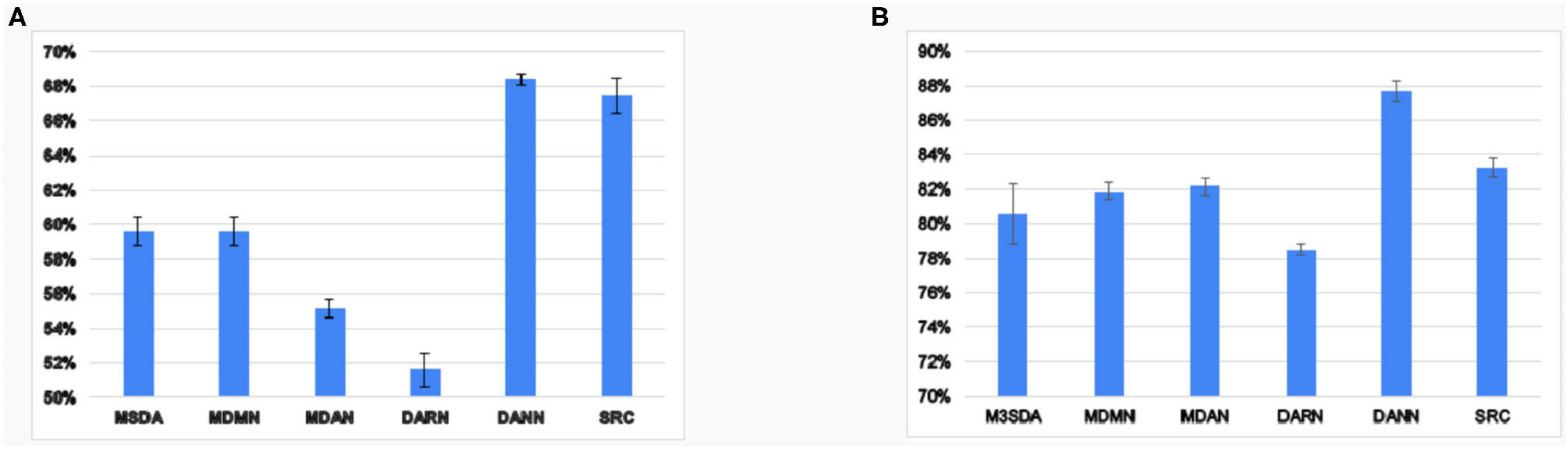
Average accuracy values 10 fold CV of site classification using latent features learned in various models for ABIDE 1**(A)** and ADHD-200**(B)**.

The previous results show that the MSDA models perform better than simply combining all the source data and utilizing it without any adaptation. To explore how well the models harmonize the source sites, we ran experiments on MSDA models' ability to make features site-invariant. Figure 5 reports the results of a two-layered fully-connected network that was trained and tested to classify the sites based on input latent features in a 10-fold CV setting. We found that, in ABIDE 1, the generalization of sites seems to be better than in the case of ADHD-200. We observe that, though MSDA methods have lower accuracy in site classification, it is still greater than chance ($\frac{1}{17}$ for ABIDE 1and $\frac{1}{8}$ for ADHD-200). This is owing to the trade-off between harmonizing sites and retaining discriminatory information for the classification that each model must tackle. Since each model tries to achieve this balance in different ways, we find that there is still some remnant site information present in the processed features by each of the MSDA techniques. Nevertheless, all MSDA methods produced latent features using which, it was difficult for the neural net to distinguish which site an instance was from. This ability to make features site-invariant is the driving force behind improving the performance with respect to baseline performance.

## 7. Conclusion

This article analyses the performance of various existing MSDA models at classifying different objectives using rs-fMRI data, using data from popular public datasets ABIDE 1 and ADHD-200. We used FCMs of data as the representative feature vector upon which the models were trained and evaluated. MSDA methods are successful in producing site-invariant latent features for the data, which in turn helps in improving classification accuracies. However, note that such methods are sensitive to the class distribution present in the data. To mitigate this, simple oversampling techniques worked well and improved the classification performances of almost all models. This can be especially useful for small and imbalanced datasets which are common in neuroscience. Furthermore, we found that some learning objectives were unaffected by MSDA architectures (e.g., age), however, this was not exhaustively tested due to data limitations. Based on the experiments conducted, we observe that M^3^SDA consistently performed well across datasets and labels and was less prone to class imbalance. Models such as DARN, MDMN, and MDAN performed better in the larger dataset—(ABIDE 1), and were sensitive to class imbalance, nevertheless, they performed significantly better when classes were balanced using simple sampling techniques. In general, we see that MDAN: only for illness classification, and M^3^SDA have improved the performance with respect to the baseline accuracies by a bigger margin than others for the majority of the classifications. Furthermore, we provide evidence that MSDA techniques are able to improve site harmonization and produce site-invariant features while extracting information that can be used for classifications. Based on these results, it is suggestive that MSDA techniques can be beneficial in improving the performance of DL techniques in neuroimaging-based applications.

## Data Availability Statement

The original contributions presented in the study are included in the article/Supplementary Material, further inquiries can be directed to the corresponding author/s.

## Author Contributions

RG and SK identified and conceptualized the research problem. RP and SK handled by along with insights from RG, RP, and SK collected and processed the neuroimaging data, which included cleaning and feature extraction processes. SK, RG, and RP designed the deep learning pipelines and conducted the experiments, and curated the results. SK, RG, and RP wrote the first draft of the paper along with SK. The draft underwent multiple revisions incorporating RG's and SK's suggestions and reviews. All authors have contributed to and have approved of the final manuscript.

## Conflict of Interest

The authors declare that the research was conducted in the absence of any commercial or financial relationships that could be construed as a potential conflict of interest.

## Publisher's Note

All claims expressed in this article are solely those of the authors and do not necessarily represent those of their affiliated organizations, or those of the publisher, the editors and the reviewers. Any product that may be evaluated in this article, or claim that may be made by its manufacturer, is not guaranteed or endorsed by the publisher.
