# Supplementary material for: Multi-Source Domain Adaptation Techniques for Mitigating Batch Effects: A Comparative Study
Source: Front Neuroinform. 2022 Apr 20;16:805117. doi: 10.3389/fninf.2022.805117 (PMC9067602; doi:10.3389/fninf.2022.805117)
Supplement: Supplementary file 1 [file Data_Sheet_1.zip › Rohan_DA_supplementary.pdf]

frontiers

Supplementary Material

1 DOMAIN ADAPTATION METHODS

In the following sub-sections, a brief description of the underlying mechanisms in each of the MSDA techniques used in this study is given.

1.1 Domain Adversarial Neural Networks (DANN)

Unlike the rest of the methods used in this study, DANN (Ajakan et al., 2014) is a single-source domain adaptation technique that has been included to compare its performance with the other methods. DANN aims to learn features which help in accurately classifying data points based on their labels while making sure that the features are domain-agnostic. The architecture of the model used for adversarial training consists of three components, first, feature extractor M_f which converts the input features to a latent representation (usually lower in dimension). The model bifurcates into two branches each of which is fed with these latent features. The first branch is the class predictor, M_c, which predicts the class to which the sample belongs to, whereas, the second branch is used for predicting in the domain of the sample and is denoted by M_d. A gradient reversal layer (GRL), $G_\mu(.)$, is attached at the start of M_d to train the model in an adversarial fashion. The loss function which is minimized is given by:

$$\begin{aligned} E(\theta_f, \theta_d, \theta_c) = \sum_{s \in [1, S-1]} \sum_{i=1}^{N_s} \mathfrak{L}_c(M_c(M_f(x_s^i; \theta_f); \theta_c), y_s^i) + \\ \sum_{k \in [1, S]} \sum_{i=1}^{N_k} \mathfrak{L}_d(M_d(G_\mu(M_f(x_k; \theta_f)); \theta_d), y_k^i) \end{aligned} \tag{S1}$$

$$\mathfrak{L}_c(\widehat{y}^i, y^i) = -y^i log(\widehat{y}^i) \tag{S2}$$

$$\mathfrak{L}_d(\widehat{y}^i, y^i) = -(y^i log(\widehat{y}^i)) + (1 - y^i) log(1 - \widehat{y}^i)) \tag{S3}$$

$$G_\mu(\mathbf{X}) = \mathbf{X}, \; \frac{dG_\mu}{d\mathbf{X}} = -\mu \mathbf{I} \tag{S4}$$

1.2 Multisource Domain Adversarial Networks (MDAN)

MDAN can be seen as a logical extension to DANN wherein multiple source domains are used instead of one single source(Zhao et al., 2018). Apart from M_f and M_c the architecture contains one domain classifier M_{d_i} for each of the $S-1$ source domains. The soft-max version of MDAN which utilizes the log-exp-max function to obtain a smoother approximation of the max function used in adversarial training was used in this work as it was shown to produce better and more computationally efficient results(Zhao et al., 2018). The model trains to minimize the following loss function:

$$E(\theta_f, \theta_{\mathbf{D}}, \theta_c) = \frac{1}{\gamma} log \sum_{s \in [1, S-1]} exp(\gamma(\mathfrak{L}_c^s + \mathfrak{L}_D^{s,S})) \tag{S5}$$

$$\alpha_s = \frac{\mathfrak{L}_c^s}{\sum_{s \in [1, S-1]} \mathfrak{L}_c^s} \tag{S6}$$

Where $\mathfrak{L}_c^s$ is the cross-entropy loss on label classification for the data from source site s and $\mathfrak{L}_D^{s,S}$ is the domain discrimination loss for data from source site s and target site S, similar to the losses defined in Eq.(S1). The values of α_s are dynamically derived during the training process using each sites' losses helping the learning happen smoothly.

1.3 Domain AggRegation Networks (DARN)

One of the main efforts in domain adaptation is during combining data from different sources, wherein, we need to select the domains which closely resemble the target domain in hand while excluding domains that are dissimilar. While inclusion of more domains provides the model with more data to train on, utilizing domains that are very different from the target domain leads to negative transfer (Jiménez-Guarneros and Gómez-Gil, 2020). DARN (Wen et al., 2020) aims at dynamically selecting and combining sites during the training phase to find the optimal selection in the trade-off between increasing sample size and decreasing negative transfer. DARN comprises of a feature extractor M_f, label classifier M_c, and a domain classifier M_{d_i} for each domain. To define the objective function of the model, first, the model losses are defined by:

$$\begin{aligned} l_s(\theta_f, \theta_c, \theta_d) = & \sum_{x^i, y^i \in D_s} \mathfrak{L}_c(M_c(M_f(x^i; \theta_f); \theta_c), y^i) + \\ & \sum_{x^i, d^i \in D_s, D_S} \mathfrak{L}_d(M_{d_s}(M_f(x^i; \theta_d); \theta_c), d^i) \end{aligned} \tag{S7}$$

Consequently the collection of these losses for all sources is $\mathbf{L} = [l_1, l_2, \cdots, l_{S-1}]^\top$. A temperature parameter τ is incorporated with the final objective defined by:

$$\min_{\boldsymbol{\alpha} \in \triangle} - < \mathbf{z}, \boldsymbol{\alpha} > + \|\boldsymbol{\alpha}\|_2 \tag{S8}$$

(define alpha's set) where $\mathbf{z} = \mathbf{L}/\tau$. To solve for the optimal $\boldsymbol{\alpha}$ values the Lagrangian dual of the above equation given by, $-\mathbf{z}^\top \boldsymbol{\alpha} + \|\boldsymbol{\alpha}\|_2 - \boldsymbol{\lambda}^\top \boldsymbol{\alpha} + \nu(\mathbf{1}^\top \boldsymbol{\alpha} - 1)$ for $\nu \in \mathbb{R}, \boldsymbol{\lambda} \geq 0$,is used. This gives us the optimal alpha values $\boldsymbol{\alpha}^*$ as:

$$\boldsymbol{\alpha}^* = \frac{[\mathbf{z} - \nu^* \mathbf{1}]_+}{\|[\mathbf{z} - \nu^* \mathbf{1}]_+\|_1} \tag{S9}$$

where ν^* is found using binary search between $[min(\mathbf{z}) - 1, max(\mathbf{z})]$. Thus, the importance of each source domain is dynamic and keeps changing throughout the training phase to find the optimal sources and their contribution in developing the features for the target domain.

1.4 Multi-Domain Matching Networks (MDMN)

MDMN also works on the concept of developing a shared feature space, however the additional step is included to improve classification performance is based on mapping the feature space distributions of every source domain among themselves as well as mapping this common feature space to the target domain. The idea is to use a domain adapter which finds the degree of similarity between all the source domains so that the strength on similarity of the target domain is shared among all the similar source domains (Li et al., 2018). The method allows all similar domains to merge together while keeping dissimilar domains away to reduce negative transfer. This is achieved by imposing a Wasserstein distance-based loss function

which encourages the features from different domains to be closer to each other. MDMN has shown to help improve the classification performance while avoiding over-fitting issues as described in Li et al. (2018). The loss function which is used for training the model is given by:

$$E(\theta_f, \theta_d) = \frac{1}{SN_0} \sum_{i=1}^{N_0} \frac{1}{N_{s_i}} \mathbf{r}_{s_i}^T \mathbf{M}_d(M_f(x_i; \theta_f); \theta_d) \tag{S10}$$

$$\mathbf{r}_s = \begin{cases} -\beta_s w_{ss'} & s' \neq s \\ \beta_s & s' = s \end{cases}, \forall s' \in [1, S] \tag{S11}$$

Where, N_{s_i} denotes the proportion of data that comes from s_i, and the data samples are taken in a mini-batch format given by $\{(\mathbf{x}_i, s_i)\} \forall i \in [1, N_0]$. In MDMN a single domain adapter is used with weight sharing instead of using S different domain adapters for computational efficiency and is denoted by, $\mathbf{M}_d(;\theta_d) = [M_{d_1}(;\theta_d), M_{d_2}(;\theta_d), \cdots M_{d_S}(;\theta_d)]$. The definitions and strategies to calculate β_s and $\mathbf{w}_s$ can be found in Li et al. (2018).

1.5 Moment Matching for MSDA (M^{3}SDA)

The main objective of M^{3}SDA is to align the target domain with the source domains while aligning the source domains among themselves simultaneously during the training process. Unlike the other methods discussed, M^{3}SDA tries to align the feature distribution moments of each source instead of using adversarial training for reducing the domain batch effects (Peng et al., 2019). One of the basic assumptions that this method is based on – is that the posterior distribution of the class labels $P_{Y|X}$ would automatically align if the model is able to align the prior feature distributions P_X of the domains. This assumption however might not hold true with practical datasets containing multiple sources. To mitigate this issue M^{3}SDA-β was introduced in Peng et al. (2019), which has been used in this study as well. M^{3}SDA-β minimizes the domain discrepancy based on the kth order cross-moment divergence denoted by $d_{CM}^k(\cdot, \cdot)$, where k is a parameter taken as an input, furthermore the training strategy utilized in Peng et al. (2019) was applied for this model. The main loss function can be written as:

$$E(\theta_f, \theta_c) = \sum_{s \in [1,S]} \sum_{i=1}^{N_s} \mathfrak{L}_d(M_c(M_f(x_s^i; \theta_f); \theta_c), y_s^i) + d_{CM}^k(F_s, F_S) \tag{S12}$$

where F_s, F_S denote the feature vectors received from M_f for the source and target data $\mathbf{x}_s$ and $\mathbf{x}_S$ respectively. Apart from the feature extractor M_f, M^{3}SDA-β also uses a pair of classifiers M_C and $M_{C'}$ for each domain denoted by $\mathbf{M}_{C'} = [(M_{C_1}, M_{C'_1}), (M_{C_2}, M_{C'_2}), \cdots, (M_{C_S}, M_{C'_S})]$. The training strategy involves using M_f and $\mathbf{M}_C$ in a three-step process wherein, first, both the models are trained together to classify multi-source samples. Next, $\mathbf{M}_C$ is trained while keeping M_f fixed to maximize the target domain discrepancy between each of the classifiers in a classifier pair from $\mathbf{M}_C$. Finally, M_f is trained while fixing $\mathbf{M}_C$ to minimize the discrepancy of each classifier pair in $\mathbf{M}_C$. The process repeats until convergence is achieved and during testing a weighted-average of the classifier outputs is used to make predictions, wherein the weights are defined using source-only accuracies as described in Peng et al. (2019).

REFERENCES

Ajakan, H., Germain, P., Larochelle, H., Laviolette, F., and Marchand, M. (2014). Domain-adversarial neural networks. *arXiv preprint arXiv:1412.4446*

Jiménez-Guarneros, M. and Gómez-Gil, P. (2020). A study of the effects of negative transfer on deep unsupervised domain adaptation methods. *Expert Systems with Applications* , 114088

Li, Y., Carlson, D. E., et al. (2018). Extracting relationships by multi-domain matching. In *Advances in Neural Information Processing Systems*. 6798–6809

Peng, X., Bai, Q., Xia, X., Huang, Z., Saenko, K., and Wang, B. (2019). Moment matching for multi-source domain adaptation. In *Proceedings of the IEEE International Conference on Computer Vision*. 1406–1415

Wen, J., Greiner, R., and Schuurmans, D. (2020). Domain aggregation networks for multi-source domain adaptation. In *International Conference on Machine Learning* (PMLR), 10214–10224

Zhao, H., Zhang, S., Wu, G., Moura, J. M., Costeira, J. P., and Gordon, G. J. (2018). Adversarial multiple source domain adaptation. *Advances in neural information processing systems* 31, 8559–8570
